# Nutritional composition and total collagen content of two commercially important edible bivalve molluscs from the Sea of Japan coast

**DOI:** 10.1007/s13197-018-3422-5

**Published:** 2018-10-01

**Authors:** Oksana Vatslavovna Tabakaeva, Anton Vadimovich Tabakaev, Wojciech Piekoszewski

**Affiliations:** 1 Department of Food Science and Technology, School of Biomedicine, Far Eastern Federal University, Vladivostok, Russia; 2 Department of Analytical Chemistry, Faculty of Chemistry, Jagiellonian University, 2 Gronostajowa Str., 30-387 Kraków, Poland

**Keywords:** Bivalve molluscs, Chemical compositions, Nutritional value, Collagen, Amino acids, Microelements

## Abstract

The study aimed to evaluate the chemical composition and nutraceutical potential of two commercially significant edible bivalve mollusc species (*Anadara broughtonii* and *Mactra chinensis*). The edible parts (motor muscle, mantle and adductor) of these molluscs were analyzed for their proximate composition, collagen content, amino acid profile, chemical score and elemental constituents. Both molluscs had low fat content (2.43–6.91 g/100 g dry weight), and protein (55.36–68.01 g/100 g dry weight) and carbohydrates (11.36–20.37 g/100 g dry weight) were their main components. Total collagen content of the edible bivalve molluscs varied from 30.5 to 39 mg/g wet weight, accounting for approximately half of their total protein content. Among amino acids, glycine, glutamate, aspartic acid, alanine, leucine, lysine and arginine were present at high levels in the edible parts of both bivalve molluscs, while the major elements present were sodium, potassium, magnesium, calcium, iron, zinc and nickel. Having high-quality protein content, edible bivalve molluscs could be excellent sources of nutritive ingredients and, after further study, may find applications in nutricosmetics and functional foods.

## Introduction

Bivalves have always been an important fishery commodity and a part of a global multi-million dollar business. They are commercially valuable products because of their high biological and nutritional value, which is associated with the presence of specific proteins and vitamins as well as their mineral composition (España et al. 2007; Karakoltsidis et al. 1995).

Bivalve molluscs are consumed as traditional food in many countries and are considered delicious and nutritious. The consumption of bivalve molluscs in some countries has increased in recent years in response to the higher market availability of aquaculture products. Various species of bivalve molluscs consumed in Russia can be produced through aquaculture or by harvesting natural resources.

Industrial-scale fishing of bivalve molluscs has developed rapidly in recent years, notably in the area of the Sea of Japan near Russia. The most important species that can be found there are *Anadara broughtonii*, *Spisula sachalinensis*, *Mactra chinensis,* and others.

*A. broughtonii* belongs to the family Arcidae and is a rather common type of Mollusca, in the Bivalvia class. It is found in the upper-sublittoral in subtropical areas of the Asian Pacific, with commercial stocks at depths of 2–15 m. The maximum lifespan of *A. broughtonii* is 65 years, the total mass of individuals varies from 80 to 380 g, and shell length is 65–80 mm. *A. broughtonii* inhabits muddy and sandy bottoms, burrowing to depths of 10–25 cm. It is found in the Yellow Sea and the Sea of Japan (Rakov 2002). *M. chinensis* from the family Mactridae—with Pacific, Asian, subtropical, and upper-sublittoral types—inhabits shallow, sandy waters (1–12 m) of the East China, Yellow, Japan and Okhotsk seas. The maximum age of this clam is up to 12 years. The total mass of individuals varies from 10 to 71 g, and shell length is 30–80 mm. The commercial size of its shell is 45 mm (Arzamastsev et al. 2001). The key macronutrients present in or seafood in general are proteins and carbohydrates. A particularly low fat content and the presence of high-quality proteins constitute nutritional properties characteristic of mollusc meat. Protein fractions and their amino acid composition are one of the most important indicators of the nutritive quality of the protein.

Despite the fact that clams have a long history of being a part of many traditional diets, data on the chemical composition of their edible parts is scarce and mostly concern only the foot, whilst the mantle and adductor can also be good sources of nutrients (Miletic et al. 1991; Orban et al. 2007; Chen et al. 2012).

The aim of this work was a comparative study of the nutritional quality of two commercially important, edible bivalve molluscs harvested on the coast of the Sea of Japan, in the Primorsky region of Russia, focusing on the total collagen content in different parts of the mollusc (muscle, mantle and adductor).

## Materials and methods

### Chemicals and reagents

All chemicals were of analytical grade. Trichloroacetic acid (TCA) was obtained from PanReac AppliChem (Darmstadt, Germany). Chloroform and methanol were obtained from Nevareactiv (St. Petersburg, Russia). Sodium hydroxide (NaOH) and potassium chloride (KCl) were purchased from Uralhiminvest (Ekaterinburg, Russia).

### Sample and preparation

The clam *A. broughtonii* ranges from 65 to 80 mm in length, and its mass varies from 80 to 200 g. The clam *M. chinensis* has a length of 45–50 mm and a mass varying from 40 to 60 g. Both mollusc species were collected once every month from Amur Bay (43°06′N and 131°44′E), Sea of Japan, in the Primorsky region in Russia in June, September and November 2013, and in February and April 2014 (all analyses were done using pooled molluscs—seasonal changes were not taken into consideration in this study). Live bivalve molluscs (about 5 kg of each species) were transported under refrigeration (+ 6 °C) to the laboratory within 3 h and sampled randomly for this study. Upon arrival, the clams were manually shucked by cutting the adductor muscle with a knife. The clam juice was removed and the edible portion, constituting approximately 10.07–12.36% of total weight for *A. broughtonii,* and 15.25–18.10% of total weight for *M. chinensis*, was collected. The edible portion was then dissected into 4 parts: muscle, mantle, adductor and viscera, which were 31.78–39.50%, 22.45–25.09%, 11.93–14.63% and 16.75–20.78% of the edible portion of *A. broughtonii*, and 27.12–30.97%, 18.68–27.16%, 9.54–12.70% and 14.80–17.35% of the edible portion of *M. chinensis*, respectively. All portions were powdered using a blender (Phillips, Guangzhou, China) in the presence of liquid nitrogen. The samples were packed in a polyethylene bag, sealed and stored at − 20 °C until use. The storage time was no longer than 1 month.

### Proximate composition analysis

The moisture content was measured according to methodology described by the Association of Official Analytical Chemists (AOAC) (2000). Samples were dried in an oven at 105 °C until a constant weight was obtained. Crude protein, fat and ash content was measured in accordance with AOAC Method 970.42 (2000). A conversion factor of 5.8 was used for determination of total crude protein in jellyfish (Doyle et al. 2006). Fat was determined according to AOAC Method 960.39 (2000). Ash was determined according to AOAC Method 920.153 (2000) by incinerating the sample in a muffle furnace at 550 °C for 12 h. Total carbohydrate was calculated by difference [100 − (moisture + protein + fat + ash)]. The obtained concentrations were expressed on a wet and dry weight basis.

### Fractionation of the edible parts of the clams

The muscle, mantle and adductor of the bivalve molluscs were fractionated in accordance with the method of Hashimoto et al. (1979). The temperature during analysis was maintained at 4 °C. The samples (20 g) were transferred to 200 mL of deionized water, and stirred using a mechanical stirrer (RZR 1 Heidolph Instruments Gmbh & Co. Schwabach, Germany) at a speed of 400 rpm for 6 h. The extract was centrifuged at 3000*g* using a centrifuge (Epp 5418R, *Eppendorf* AG, Germany) for 10 min. The process was repeated three times and the supernatants were combined. TCA was added to the solution to obtain a final concentration of 5% (weight/volume). The mixture was centrifuged at 3000 g for 10 min. The resulting precipitate was regarded as the sarcoplasmic protein fraction, and the remaining supernatant was considered as the non-protein nitrogenous compounds (NPN) fraction.

The residue from the previous extraction was stirred with 10 volumes of 0.5 M KCl (one part of residue and 10 parts of KCl solution) for 6 h, followed by centrifugation at 3000*g* for 10 min. The extraction was performed three times. The supernatants were combined and regarded as the myofibrillar protein fraction. The residue was combined with 10 volumes of 0.1 M NaOH (one part of residue and 10 parts of NaOH solution), and the mixture was stirred continuously for 6 h. The mixture was then centrifuged at 3000*g* for 10 min. The extraction was performed three times. The supernatants were combined and regarded as the alkali-soluble protein fraction. The final residue was regarded as the stroma protein fraction. The content of total nitrogen in all fractions was determined by the Kjeldahl method, AOAC Method 981.10 (2000), and after that the nitrogen distribution was calculated. The purpose of the nitrogen distribution analysis was to estimate the content of various protein fractions in different parts of bivalve molluscs, in order to further develop the technology of their use in food, and also to determine the digestibility of clam proteins. All protein fractions were also subjected to determination of protein patterns.

### Amino acid analysis

The frozen portions of the clams were hydrolyzed for 22 h at 110 ± 1 °C with 6 M HCl in sealed glass tubes filled with nitrogen. Following hydrolysis, 1 mL of hydrolyzate was withdrawn and evaporated to dryness under vacuum at 45 °C to remove HCl. The hydrolyzate was dissolved in 1 mL of sodium citrate buffer (pH 2.2), and then the samples were analyzed by a Hitachi L8800 Automatic Amino Acid Analyzer (Hitachi, Tokyo, Japan). The identity and quantity of each amino acid was assessed by comparison with the retention time and peak area of a standard (Sigma).

The tryptophan content was determined in a separate analysis. The weighed samples were hydrolyzed in 5 N NaOH containing 5% SnCl2 (w*/*v) for 20 h at 110 °C (Hugli and Moore 1972). After hydrolysis, the hydrolyzate was neutralized with 6 N HCl and centrifuged, and then the supernatant was analyzed by a Hitachi L8800 Automatic Amino Acid Analyzer. The identity and quantity of tryptophan was assessed by comparison with the retention time and peak area of a standard (Sigma). All determinations were performed in triplicate. Amino acid ((threonine (Thr), tryptophan (Trp), cysteine + methionine (Cys + Met), valine (Val), phenylalanine + tryptophan (Phe + Tyr), isoleucine (Ile), leucine (Leu), and lysine (Lys)) contents were expressed as mg/g wet weight.

### Amino acid scores

The essential amino acid score was calculated in relation to the FAO*/*WHO reference amino acid requirement pattern for preschool and school children (3–10 years old) and adults (WHO/FAO/UNU Expert Consultation 2007):

$$ {\text{Amino}}\;{\text{acid}}\;{\text{score}} = {\text{Amino}}\;{\text{acid}}\;{\text{in}}\;{\text{a}}\;{\text{sample/reference}}\;{\text{amino}}\;{\text{acid}} * 100 $$

### Total collagen content

Total collagen content can be extrapolated by multiplying the amount of total hydroxyproline content in each sample by a factor of 8. The concentration of hydroxyproline was determined after acid hydrolysis of samples using 6 M HCl for 6 h at 105 °C. The hydroxyproline (Hyp) content was quantified using a commercial Hyp detection kit. The OD values of the samples were measured at 550 nm using a hydroxyproline colorimetric assay kit (BioVision, BioVision Inc, CA, USA) (Woessner 1961).

Total collagen content in samples expressed in g/100 g dry weight was estimated as follow:

$$ {\text{Total collagencontent}}\,(\% {\text{dv}}) = {\text{H}} \times {\text{CF}} $$

where H = total hydroxyproline content (g/100 g dry weight), CF = conversion factor.

Meanwhile, the ratio of total collagen to total protein content was estimated as follows:

$$ {\text{Total}}\;{\text{collagen}}\,(\% ) = {\text{Estimated}}\;{\text{collagen}}\;{\text{content/Total}}\;{\text{protein}}\;{\text{content}} \times 100 $$

where,

$$ {\text{Estimated}}\;{\text{collagen}}\;{\text{content}}\,\left( {{\text{g/}}1000\,{\text{g}}} \right) = {\text{Hyp}}\;{\text{content}}({\text{g/}}1000\,{\text{g}}) \times 8 $$

$$ {\text{Total}}\;{\text{protein}}\;{\text{content}} = {\text{amino}}\;{\text{acids}}\;{\text{content}}\;\left( {{\text{including}}\;{\text{hydroxyproline}}} \right)\;\left( {{\text{g/}}1000\,{\text{g}}} \right) $$

The conversion factor used to convert Hyp content for the estimation of total collagen content in this study was 8 as collagen has been reported to contain 12.5%–13.5% Hyp, depending on the conversion factor used in the conversion of nitrogen content to protein (Ignateva et al. 2007; Kolar 1990; Mazorra-Manzano et al. 2012). Previously, in the case of animal skins, e.g. fish, which contained high protein, a conversion factor of 7.5 or 7.7 was applied (bigeye snapper skin) (Kittiphattanabawon et al. 2005); 13.5 (murine lungs) (Kliment et al. 2011), and in some cases 14.7 (Baltic cod) (Sadowska et al. 2003) for the estimation of collagen based on Hyp content.

### Determination of elements

Mineral contents in the dried clam portions were determined by atomic absorption spectrometry using an AA-7000 spectrometer (Shimadzu, Japan) with the system of double atomization (flame and electrothermal). Iron (Fe), zinc (Zn), copper (Cu), manganese (Mn), nickel (Ni), molybdenum (Mo), aluminium (Al), cadmium (Cd), lead (Pb) and chromium (Cr) were determined using a graphite cuvette and deuterium background corrector lamp according to the AOAC method (2000).

The content of mercury was determined by flameless atomic absorption using the “Hg-1” mercury analyzer (« Hiranuma » , Japan).

Determination of sodium (Na), potassium (K), calcium (Ca) and magnesium (Mg) in the dried clam portions was performed using an air-acetylene flame at a fuel flow rate of 50 L h^−1^ according to AOAC Method 999.11 (2000). The burner height was 5–12, 4–10 and 4–10 mm; slit width was 0.2, 1.0, 0.5 and 0.5 nm; the wavelength was 589.0, 766.5, 422.7 and 258.2 nm; and the lamp current was 6.0, 4.0, 5.0 and 5.0 mA for Na, K, Ca and Mg, respectively. Five specimens, which were previously dried at 80 °C, from five different molluscs were combined into one sample after digestion with nitric acid. Limits of detection and quantification for the investigated elements are shown in Table 1.

**Table 1:** Limit of detection and limit of quantification of the method for determined metals (μg g^−1^)

Minerals	LOD	LOQ
Na	7.00	20.55
K	5.00	16.80
Ca	8.00	20.46
Mg	5.00	13.45
Fe	0.50	1.86
Zn	0.09	0.21
Mn	0.20	0.74
Cu	0.05	0.17
Cr	0.02	0.05
Ni	0.10	0.28
Mo	0.03	0.08
Al	0.10	0.30
Pb	0.07	0.23
Cd	0.05	0.16
Hg	0.04	0.12

*LOD* limit of detection, *LOQ* limit of quantification

The limits of detection (LOD) (calculated as 3 s/a, where ‘a’ is the slope of the calibration curve and ‘s’ is the standard deviation of 10 consecutive measurements of the blank solution) were between 0.01 and 7 μg g^−1^. The limits of quantification (LOQ) (calculated as 10 s/a, where ‘a’ is the slope of the calibration curve and ‘s’ is the standard deviation of 10 consecutive measurements of the blank solution) were between 0.05 and 20.55 μg g^−1^.

### Statistical analysis

The results were expressed as mean values with standard deviation (*n *= 3). The differences between the mean values for the three parts of each bivalve mollusc were calculated using one-way analysis of variance (ANOVA), and statistically significant differences were reported at *p *< 0.05. Data analysis was carried out using SPSS 16 software (SPSS Inc., Chicago, IL, USA).

## Results and discussion

### Proximate composition of the edible parts of the molluscs

In general, the edible parts of the bivalve molluscs *M. chinensis* and *A. broughtonii* were rich in protein and carbohydrates, and had high moisture levels (Table 2).

**Table 2:** Proximate composition (g/100 g) muscle, mantle and adductor clams

Compositions (%)	*M. chinensis*			*A. broughtonii*		
	Muscle	Mantle	Adductor	Muscle	Mantle	Adductor
*Of wet mass*						
Moisture	80.16 ± 1.06	82.32 ± 0.75	81.35 ± 0.84	78.55 ± 1.16	80.70 ± 1.14*	78.94 ± 1.01
Protein	14.55 ± 0.31	12.20 ± 0.29*	13.13 ± 0.37*	16.50 ± 0.41*	13.14 ± 0.35	15.82 ± 0.33
Ash	1.89 ± 0.05*	1.45 ± 0.02	1.61 ± 0.04	1.16 ± 0.03	1.06 ± 0.02	0.95 ± 0.02
Fat	0.53 ± 0.01	0.43 ± 0.00	0.73 ± 0.02	1.12 ± 0.04	0.87 ± 0.03	1.37 ± 0.04*
*Of dry mass*						
Moisture	10.04 ± 0.22	13.64 ± 0.21*	12.77 ± 0.20	10.48 ± 0.18*	11.98 ± 0.16	11.14 ± 0.15
Protein	62.71 ± 0.96	55.36 ± 0.82*	57.63 ± 1.02	68.01 ± 1.12	57.41 ± 0.95*	60.19 ± 1.03*
Ash	9.45 ± 0.17*	8.20 ± 0.12*	5.63 ± 0.10	5.41 ± 0.09	6.49 ± 0.11	4.53 ± 0.07
Fat	2.65 ± 0.02*	2.43 ± 0.01*	6.91 ± 0.09	5.22 ± 0.06	4.51 ± 0.04	6.52 ± 0.07
Carbohydrate	15.15	20.37	17.06	11.36	19.67	17.62

Data are mean ± standard deviation (n = 3). Values in the parenthesis represent the content based on dry weight

Statistically significant difference *A. broughtonii* versus *M. chinensis*; *p* < 0.05; n = 3

Crude protein = %N × 5.80 (Doyle, Houghton, McDevitt, Davenport, & Hays, 2007)

Carbohydrate = 100% − R% (moisture + protein + fat + ash)

Such composition is typical of other bivalve molluscs (Karnjanapratum et al. 2013). It was established that the moisture content of the clams’ tissues changed from 81 g/100 g wet weight to 13.42 g/100 g dry weight. Similar values were reported by Oliveira et al. (2011).

The edible parts of *M. chinensis* were characterized by a higher moisture content than those of *A. broughtonii*. The mantle of *A. broughtonii* and *M. chinensis* had the highest level of moisture in comparison to other parts. The content of basic components in the raw edible parts was identical for both species and decreased in the following order: moisture > proteins > carbohydrates > ash > lipids. The content of proteins, carbohydrates and ash was higher in the parts of *M. chinensis* in comparison to *A. broughtoni*.

The protein content in various parts of the molluscs decreased in the following order: muscle > adductor > mantle. Differences in the total protein content between the muscle and the mantle was 19.3% (wet weight) for *M. chinensis,* and 25.6% (wet weight) for *A. broughtonii*. These findings were consistent with the results reported by other researchers for protein and moisture content for bivalve molluscs. For instance, the moisture of the Asian hard clams *Meretrix meretrix* (Xie et al. 2012) and *Meretrix lusoria* (Karnjanapratum et al. 2013) was 78.33–81.12% and 76.23–84.22%, respectively.

The distribution of total minerals content in the edible parts of the mollusc species was different. The highest content of ash was observed in the muscle of *M. chinensis*, while in the case of *A. broughtonii*—in the mantle. Moreover, the quantitative differences in the level of elements between *A. broughtonii* and *M. chinensis* reached statistical significance (*p *< 0.05) and were equal to 36.8% for the mantle, 62.9% for muscle and 69.5% for adductor. The lowest ash content was observed for adductor of *A. broughtonii*. It was established that all edible parts of the clams, regardless of the species, contained a small amount of lipids not exceeding 1.37%, which was in accordance with findings from previous studies on the bivalve molluscs (Chakraborty et al. 2002; Fuentes et al. 2009; Karnjanapratum et al. 2013). It was found that the tissues of *M. chinensis* had less fat content than those of *A. broughtonii* (*p *< 0.05). The distribution of fat between the individual edible parts was similar in both clam species, and its level decreased in the following order: adductor > muscle > mantle. The fat content in the *A. broughtonii* adductor was almost two times higher than in the same part of *M. chinensis*. It is well known that the fat of marine organisms, including bivalve molluscs, is rich in biologically active polyunsaturated fatty acids—eicosapentaenoic acid (C20:5 n-3, EPA) and docosahexaenoic acid (C22:6 n-3, DHA), which makes the lipids in the molluscs (despite their low content) valuable for the food industry (Fernández-Reiriz et al. 2006; Kawashima and Ohnishi 2014; Saito 2014).

The chemical composition of the organs and tissues of the molluscs depends on age, sex, sex maturity ratings, water temperature, stomach fullness, stress level and other environmental factors (Kasyanov 1989). The variety of functions of individual parts and tissues may also be linked to significant differences in their mineral content (Gosling 2003).

### Distribution of nitrogenous constituents in the clams *A. broughtonii* and *M. chinensis*

The fractional protein composition of the edible parts of the molluscs (Table 3) was significantly different.

**Table 3:** Nitrogen distribution in the muscle, mantle and adductor of clams *A. broughtoni* and *M. chinensis*

Nitrogen distribution (%)	*M. chinensis*			*A. broughtoni*		
	Muscle	Mantle	Adductor	Muscle	Mantle	Adductor
Non protein nitrogen	15.20 ± 0.27	9.05 ± 0.14	12.01 ± 0.10	19.50 ± 0.13	11.23 ± 0.08	14.91 ± 0.12
Sarcoplasmic	33.10 ± 0.93	37.44 ± 0.84*	30.18 ± 0.35	28.71 ± 1.01*	25.14 ± 0.69*	29.40 ± 1.10*
Myofibrillar	23.54 ± 0.01*	25.16 ± 2.13	29.33 ± 1.85	32.16 ± 0.94*	24.58 ± 0.51	36.84 ± 1.27*
Alkali	8.56 ± 0.09	10.29 ± 0.09*	11.97 ± 0.09*	13.64 ± 0.09*	20.37 ± 0.23	18.98 ± 0.08
Stroma	30.90 ± 0.02	28.67 ± 1.13	18.40 ± 0.96*	25.66 ± 1.03*	30.18 ± 0.19*	14.97 ± 0.40

Data are mean ± standard deviation (n = 3)

Statistically significant difference *A. broughtonii* versus *M. chinensis*; *p < 0.05; n = 3

In general, proteins were characterized by high content of sarcoplasmic and alkali fractions (*p *< 0.05).

More myofibrillar proteins were observed for *A. broughtonii* than *M. chinensis*. Myofibrillar proteins in the edible parts of the molluscs changed in the following, order: adductor > mantle > muscle. There were no statistically significant difference in the content of stroma proteins between the two mollusc species and their individual parts (*p *> 0.05). Stroma proteins were the predominant proteins in the muscle and mantle of the molluscs, sarcoplasmic proteins were prevalent in the adductor of *M. chinensis*, while myofibrillar proteins were predominant in the adductor of *A. broughtonii*. The content of myofibrillar proteins varied greatly between the mollusc species—from 23.5% in the muscle of *M. chinensis* to 36.8% in the adductor of *A. broughtonii*. Sarcoplasmic proteins in fish and molluscs are mainly myoglobin, enzymes and other albumins (Hui et al. 2012).

### Amino acid composition

All edible parts of the molluscs showed high content of amino acids. Regardless of the body part, the major amino acids were aspartic acid, glutamic acid, glycine, alanine and leucine. In general, the adductor was characterized by higher content of all amino acids than other edible parts of the clams (*p *< 0.05).

The total amino acid content in the mantle (89.03 mg/g wet weight of *M. chinensis* and 75.63 mg/g wet weight of *A. broughtonii*) differed significantly between the two species and was equal to 17.7%. There were no statistical differences in the total content of amino acids in the muscle (88.31 mg/g wet weight of *M. chinensis* and 87.11 mg/g wet weight of *A. broughtonii*) between the two clam species (*p *> 0.05). The content of cysteine, methionine, alanine and glutamic acid in the muscle of the molluscs was significantly different when comparing the two clam species. The differences were bigger in the mantle and related to the content of aspartic and glutamic acids, threonine, serine, proline, glycine, cysteine, lysine and arginine. The mantle of *M. chinensis* contained much more of these amino acids than that of *A. broughtonii*. Differences in the content of amino acids in the adductor between the two mollusc species were statistically insignificant and concerned only serine, isoleucine, and tryptophan.

**Fig. 1:**
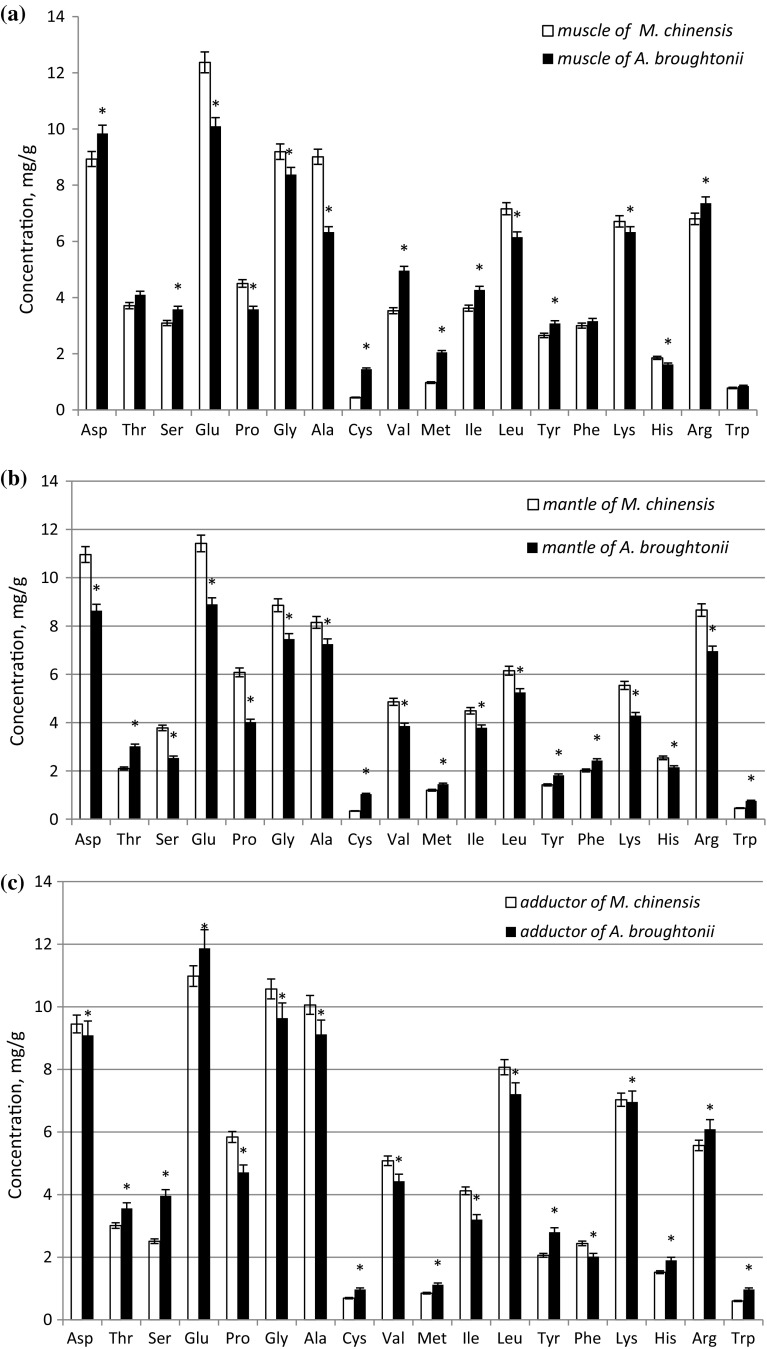
Amino acids composition (mg/g wet weight) of different parts: **a** muscle, **b** mantle and **c** adductor of clams *A. broughtonii* and *M. chinensis*/data are represented as the mean ± SD (One-way ANOVA followed by Tukey post hoc test). *Statistically significant difference *A. broughtonii* versus M*. chinensis*; *p* < 0.05; n = 3

The proportions of the major groups of amino acids—essential (isoleucine, leucine, lysine, methionine, cysteine, phenylalanine, tyrosine, threonine, valine, and tryptophan), conditionally essential (histidine, arginine, glycine, proline, and serine) and non-essential (alanine, glutamic and aspartic acid)—varied between the two investigated clam species. The level of essential amino acids ranged from 32.1% of the total content of amino acids in the mantle of *M. chinensis* to 41.8% in the muscle of *A. broughtonii* (Fig. 1). The adductor of the molluscs was characterized by an identical level of essential amino acids. The contents of conditionally essential and non-essential amino acids were not significantly different (*p *> 0.05). The content of essential acids in all parts of the investigated mollusks (except for the mantle of *M. chinensis*) was high. Overall, the differences in the level of non-essential amino acids between the two clam species were not very high.

### Amino acid scores

Amino acid scores and the first limiting amino acid are established methods of assessing the quality of proteins, which are widely used in studies on the nutritive value of proteins (Friedman 1996; WHO/FAO/UNU Expert Consultation 2007).

The results of the evaluation of amino acid scores for various edible parts of molluscs are presented in Table 4.

**Table 4:** Amino acid scores of different food parts clams. *Source*: Reference amino acid pattern of adults (FAO*/*WHO*/*UNU 2007)

Amino acid	*M. chinensis* (mg*/*g protein)			*A. Broughtonii* (mg*/*g protein)			Reference	Score *M. chinensis*			Score *A. broughtonii*		
	Muscle	Mantle	Adductor	Muscle	Mantle	Adductor		Muscle	Mantle	Adductor	Muscle	Mantle	Adductor
Thr	32.0 ± 1.1	30.1 ± 1.3	25.2 ± 1.0	34.1 ± 1.3	33.0 ± 1.2	28.1 ± 1.0	23	139	130	109	148	143	122
Trp	8.1 ± 0.2	8.0 ± 0.2	7.0 ± 0.1	9.0 ± 0.3	9.0 ± 0.4	10.0 ± 0.4	6	133	133	117	150	150	167
Cys + Met	16.0 ± 0.6	20.1 ± 0.7	24.1 ± 1.0	31.1 ± 1.1	28.1 ± 1.0	20.1 ± 0.7	22	73	91	109	141	127	91
Val	37.0 ± 1.4	48.1 ± 2.1	35.0 ± 1.3	50.0 ± 2.0	40.1 ± 1.7	33.0 ± 1.1	39	95	123	90	128	103	85
Phe + Tyr	34.2 ± 1.5	49.0 ± 2.0	50.1 ± 2.1	53.0 ± 2.1	55.1 ± 2.1	44.0 ± 1.8	38	89	129	132	139	145	116
Ile	30.1 ± 1.0	35.0 ± 1.4	37.0 ± 1.2	43.0 ± 1.7	32.0 ± 1.0	31.1 ± 1.0	30	100	117	123	143	107	103
Leu	81.0 ± 3.2	75.0 ± 3.0	79.0 ± 3.0	72.1 ± 2.9	69.0 ± 2.7	70.0 ± 2.6	59	137	127	134	122	117	119
Lys	76.0 ± 3.1	70.0 ± 3.0	80.0 ± 3.5	74.0 ± 3.0	64.1 ± 2.6	74.0 ± 2.9	45	169	156	178	164	142	164
Total	313.4 ± 14.2	335.3 ± 15.5	337.4 ± 15.4	366.3 ± 16.3	340.4 ± 15.1	310.3 ± 14.3	262						

Data are mean ± standard deviation (n = 3)

The total essential amino acids content in the individual parts of the clams was higher than the recommended daily intake (262 mg/g total protein) and varied from 310 mg/g total protein for adductor to 366 mg/g total protein for mantle. The muscle and mantle of *A. broughtonii* were characterized by higher essential amino acids than the same parts of *M. chinensis.* A comparison of the amino acid score of proteins in *M. chinensis* and *A. broughtonii* showed that there was a distinct difference between them. The proteins in the edible parts of *M. chinensis* were characterized by 3 limiting amino acids, while the proteins in *A. broughtonii* had 2 limiting amino acids.

Then, comparing our results with the amino acid composition of the reference protein recommended by FAO/WHO/UNU, it was found that almost all the main indicators of the amino acids in the parts of the analysed clams were higher than 100%, with the exception of cysteine + methionine and valine. Therefore, cysteine + methionine and valine are the limiting amino acids. Although direct hydrolysis may have resulted in about 10% loss of methionine and 55% loss of cysteine (Xie et al. 2012; Spindler et al. 1984), the content of the sulphur-containing amino acids (cysteine and methionine) exceeded 100 in all the samples. Thus, the proteins of the molluscs *A. broughtonii* and *M. chinensis* are rich in the S-containing amino acids.

### Total hydroxyproline and collagen content

The evaluation of the collagen content was carried out on the basis of analysing the hydroxyproline content. All parts of the investigated mollusks were characterized by a high content of hydroxyproline, and the highest value was observed for *M. chinensis*. The mantle of *M. chinensis* (98.8 mg/g dry weight) and *A. broughtonii* (90.0 mg/g dry weight) contained more hydroxyproline (*p *< 0.05) than the muscle (87.5 mg/g and 85.0 mg/g dry weight, respectively) and adductor (83.8 mg/g and 76.3 mg/g dry weight, respectively). The hydroxyproline content of the edible parts of molluscs was comparable to level for jellyfish reported earlier (Khong et al. 2016).

The conversion factor used to convert the hydroxyproline content for the estimation of total collagen content in this study was 8.07, as collagen has been reported to contain about 12.5%–13.5% hydroxyproline, depending on the conversion factor used in the conversion of nitrogen content to protein content (Ignateva et al. 2007; Kolar 1990; Mazorra-Manzano et al. 2012). Overall, the trend for estimated total collagen in the molluscs was the same as for hydroxyproline content. Among the edible parts, the highest content of total collagen was found in the mantle (*p *< 0.05), and it was statistically higher in *M. chinensis* than in *A. broughtonii.* Similarly in adductor concentration of total collagen was statistically higher in *M. chinensis* than in *A. broughtonii.* The collagen level in the muscle of both of the species of molluscs varied insignificantly (*p *> 0.05), but the muscle was characterized by a higher content than the adductor. The ratio of collagen to total protein content (collagen content/crude protein) was not significantly different between the investigated clam species as well as between their individual edible parts (*p *> 0.05). The proteins in the adductor of *M. chinensis* contained less collagen (30.5%) than the proteins in the mantle of *A. broughtonii* (38.8%). Collagen is therefore the major protein in the edible parts of the analysed molluscs.

### Mineral content

Table 5 shows the level of elements in the tissues of *M. chinensis* and *A. broughtonii*.

**Table 5:** Mineral contents of the edible parts of clams *A. broughtonii* and *M. chinensis*

Minerals	*A. broughtonii*			*M. chinensis*		
	Mantle	Muscle	Adductor	Mantle	Muscle	Adductor
Dry weight (mg 100 g^−1^)						
Na	1393 ± 29.7*	1527 ± 30.4	1353 ± 25.7*	1134 ± 31.1*	1307 ± 40.5	1490 ± 27.7
K	1088 ± 30.1	1905 ± 32.9**	1478 ± 31.6	1216 ± 28.1	2293 ± 45.1*	1742 ± 29.8**
Ca	594.3 ± 10.7**	1550 ± 20.6*	790.6 ± 15.6**	803.4 ± 17.9	945.9 ± 20.6	1067 ± 28.4*
Mg	453.5 ± 9.8	507.4 ± 10.7*	419.3 ± 7.9	524.6 ± 10.2**	395.3 ± 7.4	387.9 ± 7.8
Dry weight (μg 100 g^−1^)						
Fe	42.69 ± 1.01**	48.43 ± 1.14*	26.35 ± 0.33	50.45 ± 0.78	39.96 ± 0.45**	35.72 ± 0.63*
Zn	21.24 ± 0.11	20.12 ± 0.10*	18.54 ± 0.09*	15.06 ± 0.08*	17.60 ± 0.10*	17.93 ± 0.31*
Mn	11.23 ± 0.07**	9.60 ± 0.05	7.87 ± 0.04	8.70 ± 0.06	11.91 ± 0.07*	10.37 ± 0.20
Cu	0.35 ± 0.00*	0.47 ± 0.02**	0.18 ± 0.00	0.21 ± 0.00**	0.25 ± 0.00	0.16 ± 0.00**
Cr	0.12 ± 0.00*	0.13 ± 0.00*	0.08 ± 0.00	0.10 ± 0.00	0.11 ± 0.00	0.05 ± 0.00**
Ni	52.09 ± 1.10**	68.70 ± 1.54*	56.45 ± 0.90	70.56 ± 0.97	49.31 ± 0.59**	55.42 ± 0.78*
Mo	0.19 ± 0.00*	0.18 ± 0.00*	0.09 ± 0.00	0.15 ± 0.00	0.17 ± 0.00	0.08 ± 0.00**
Al	0.56 ± 0.01*	0.60 ± 0.02*	0.41 ± 0.01	0.50 ± 0.01*	0.65 ± 0.03	0.30 ± 0.01**
Pb	0.25 ± 0.00*	< LOQ	< LOQ	< LOQ	< LOQ	< LOQ
Cd	< LOQ	< LOQ	< LOQ	< LOQ	< LOQ	< LOQ
Hg	< LOQ	0.13 ± 0.00	< LOQ	0.17 ± 0.00**	0.12 ± 0.00	< LOQ

*LOQ* limit of quantification

Data are mean ± standard deviation (n = 3)

Statistically significant difference *A. broughtonii* versus *M. chinensis*; *p < 0.05;* p* < 0.01; n = 3

Na (1134–1527 mg 100 g^−1^) and K (1088–2293 mg 100 g^−1^) were the main macroelements in all of the clam parts, and the highest content of Na was observed for muscle of *A. broughtonii* and adductor of *M. chinensis*, while the highest content of K was observed for muscle of *A. broughtonii* and *M. chinensis*. The highest Ca content was observed for muscle of *A. broughtonii* and for adductor of *M. chinensis*. Ca and K content were significantly different for two species of the molluscs (*p *< 0.05).

The major microelements present in the tissues of the clams were Fe, Zn, Cr, Ni and Mn. Other detected elements were Cu, Cr, Mo and Al, of which Cu and Al showed the highest concentrations. The tissues of *M. chinensis* were characterized by the highest content of Fe, Ni, Al and Mn, while the tissues of *A. broughtonii* contained more Zn, Cu, Cr, and Mo.

## Conclusion

The edible molluscs contained high amount of water, whereas the dry mass was rich in protein and minerals, but low in fats. The tissues of *A. broughtonii* contained more protein and fat than those of *M. chinensis*, whereas ash content was significantly higher in the tissues of *M. chinensis* than those of *A. broughtonii*. The major elements present in the mollusc tissues were sodium, potassium, magnesium, calcium, iron and zinc. Collagen was found to be the major protein in edible molluscs, and aspartic acid, glutamic acid, glycine, alanine and leucine were found to be the dominant amino acids. Protein was found to be significantly higher in the mantle compared to the muscle and adductor, whereas ash content was significantly higher in the muscle than the mantle and adductor Na, K, Mg Ca, Fe and Z were major elements in mollus.

Being high in protein (especially collagen) and mineral edible molluscs has potentially for the development of novel nutraceutical, nutricosmetics and functional foods.

## References

[CR1] AOAC (2000). Official method of analytical chemists.

[CR2] Arzamastsev IS, Yakovlev YuM, Evseev GA (2001). Atlas invertebrates and seaweed of the seas of the Far East of Russia.

[CR3] Chakraborty S, Ghosh S, Bhattacharyya DK (2002). Lipid composition of bivalve *Pelecyora trigona*. J Food Sci Technol.

[CR4] Chen DW, Su J, Liu X-L, Dong-Mei Y, Ying L, Wei-Ming J (2012). Amino acid profiles of bivalve molluscs from Beibu Gulf, China. J Aquat Food Prod Technol.

[CR5] Doyle TK, Houghton JD, McDevitt R, Davenport J, Hays DC (2006). The energy density of jellyfish: estimates from bomb-calorimetry and proximate composition. J Exp Mar Biol Ecol.

[CR6] España MSA, Rodríguez EM, Romero CD (2007). Comparison of mineral and trace element concentrations in two molluscs from the Strait of Magellan (Chile). J Food Compos Anal.

[CR7] Fernández-Reiriz MJ, Labarta U, lbentosa M M, Pérez-Camacho A (2006). Lipid composition of *Ruditapes philippinarum* spat: effect of ration and diet quality. Comp Biochem Physiol B Biochem Mol Biol.

[CR8] Friedman M (1996). Nutritional value of proteins from different food sources. J Agric Food Chem.

[CR9] Fuentes I, Fernandez-Segovia JA, Escriche Serra JA (2009). Comparison of physico-chemical parameters and composition of mussels (*Mytilus galloprovincialis* Lmk.) from different Spanish origins. Food Chem.

[CR10] Gosling EM (2003). Bivalve molluscs: biology, ecology and culture.

[CR11] Hashimoto K, Watabe S, Kono M, Shiro K (1979). Muscle protein composition of sardine and mackerel. Nippon Suisan Gakk.

[CR38] Hugli TE, Moore S (1972). Determination of the tryptophan content of proteins by ion exchange chromatography of alkaline hydrolysates. J Biol Chem.

[CR12] Hui YH, Gross NH, Kristinsson G, Lim MH, Nip WK, Siow LF, Simpson BK (2012). Chapter 19: Biochemistry of seafood processing. Food biochemistry and food processing.

[CR13] Ignateva NY, Danilov N, Averkiev S, Obrezkova M, Lunin V (2007). Determination of hydroxyproline in tissues and the evaluation of the collagen content of the tissues. J Anal Chem.

[CR14] Karakoltsidis PA, Zotos A, Constantinides SM (1995). Composition of the commercially important Mediterranean finfish, crustaceans and molluscs. J Food Compos Anal.

[CR15] Karnjanapratum S, Benjaku S, Soottawat K, Hideki T, Tassi YH (2013). Chemical compositions and nutritional value of Asian hard clam (*Meretrix lusoria*) from the coast of Andaman Sea. Food Chem.

[CR16] Kasyanov VL (1989). Reproductive strategy of sea two-fold molluscs and erinaceouses.

[CR17] Kawashima H, Ohnishi M (2014). Identification of minor fatty acids and various nonmethylene interrupted diene isomers in mantle, muscle, and viscera of the marine bivalve *Megangulus zyonoensis*. Lipids.

[CR18] Khong NMH, Yusoff FMd, Jamilah B, Basri M, Maznah I, WeiChan K (2016). Nutritional composition and total collagen content of three commercially important edible jellyfish. Food Chem.

[CR19] Kittiphattanabawon P, Benjakul S, Visessanguan W, Nagai T, Tanaka M (2005). Characterisation of acid-soluble collagen from skin and bone of bigeye snapper *Priacanthus tayenus*. Food Chem.

[CR20] Kliment CR, Englert JM, Crum LP, Oury TD (2011). A novel method for accurate collagen and biochemical assessment of pulmonary tissue utilizing one animal. Int J Clin Exp Pathol.

[CR21] Kolar K (1990). Colorimetric determination of hydroxyproline as measure of collagen content in meat and meat products: NMKL collaborative study. J Assoc Anal Chem.

[CR22] Mazorra-Manzano MA, Torres-Llanez MJ, González-Córdova AF, Vallejo-Cordoba B (2012). A capillary electrophoresis method for the determination of hydroxyproline as a collagen content index in meat products. Food Anal Met.

[CR23] Method 920.153 AOAC (2000). Official method of analytical chemists.

[CR24] Method 960.39, AOAC (2000). Official method of analytical chemists.

[CR25] Method 970.42, AOAC (2000). Official method of analytical chemists.

[CR26] Method 981.10, AOAC (2000). Official method of analytical chemists.

[CR27] Method 999.11, AOAC (2000). Official method of analytical chemists.

[CR28] Miletic I, Miric M, Lalic Z, Sobajic S (1991). Composition of lipids and proteins of several species of molluscs, marine and terrestrial, from the Adriatic Sea and Serbia. Food Chem.

[CR29] Oliveira CM, Bechtel PJ, Nguyen DX, Gurer L, Crapo CA, Fong Q (2011). Chemical composition and texture of commercial geoduck clams (*Panopea abrupta*) harvested in Southeast Alaska. J Shellfish Res.

[CR30] Orban E, Di Lena G, Nevigato T, Casini I, Caproni R, Santaroni G (2007). Nutritional and commercial quality of the striped venus clam, *Chamelea gallina*, from the Adriatic sea. Food Chem.

[CR31] Rakov VA (2002). Determinants of two-fold molluscs of Primorsky Krai.

[CR32] Sadowska M, Kołodziejska I, Niecikowska C (2003). Isolation of collagen from the skins of Baltic cod (*Gadus morhua*). Food Chem.

[CR33] Saito H (2014). Lipid and FA composition of the pearl oyster *Pinctada fucata martensii*: influence of season and maturation. Lipids.

[CR34] Spindler M, Stadler R, Tanner H (1984). Amino acid analysis of feedstuffs: determination of methionine and cystine after oxidation with performic acid and hydrolysis. J Agric Food Chem.

[CR35] WHO/FAO/UNU Expert Consultation (2007). Proteins and amino acids in human nutrition.

[CR36] Woessner JF (1961). The determination of hydroxyproline in tissue and protein samples containing small proportions of this imino acid. Arch Biochem Biophys.

[CR37] Xie W, Chen C, Lui X, Wang B, Sun U, Yan M (2012). Meretrix meretrix: activity components and their bioactivities. Life Sci J.

